# Low Content of Cyclosporine A and Its Metabolites in the Colostrum of Post-Transplant Mothers

**DOI:** 10.3390/nu12092713

**Published:** 2020-09-04

**Authors:** Bożena Kociszewska-Najman, Natalia Mazanowska, Beata Borek-Dzięcioł, Leszek Pączek, Emilia Samborowska, Monika Szpotańska-Sikorska, Bronisława Pietrzak, Michał Dadlez, Mirosław Wielgoś

**Affiliations:** 1 Department of Neonatology, Medical University of Warsaw, 02-091 Warsaw, Poland; bnajman@wum.edu.pl (B.K.-N.); beataborek777@gmail.com (B.B.-D.); 2 First Department of Obstetrics and Gynecology, Medical University of Warsaw, 02-015 Warsaw, Poland; mszpotanska@wp.pl (M.S.-S.); bpietrzak@wum.edu.pl (B.P.); miroslaw.wielgos@wum.edu.pl (M.W.); 3 Department of Immunology, Transplant Medicine and Internal Diseases, Transplantation Institute, Medical University of Warsaw, 02-014 Warsaw, Poland; leszek.paczek@wum.edu.pl; 4 Department of Bioinformatics, Institute of Biochemistry and Biophysics, Polish Academy of Sciences, 02-106 Warsaw, Poland; 5 Mass Spectrometry Laboratory, Institute of Biochemistry and Biophysics, Polish Academy of Sciences, 02-106 Warsaw, Poland; emi.sambor@gmail.com (E.S.); michald@ibb.waw.pl (M.D.); 6 Institute of Genetics and Biotechnology, Biology Department, Warsaw University, 02-106 Warsaw, Poland

**Keywords:** breastfeeding, immunosuppression, cyclosporine A, transplant, lactation

## Abstract

The rate of post-transplant mothers who breastfeed while on immunosuppression is progressively increasing. Data on breastfeeding while on cyclosporine-based regimens are limited. Therefore, we assessed the amount of cyclosporine and its metabolites that might be ingested by a breastfed infant by measuring the concentration of cyclosporine and its metabolites in the colostrum of seven post-transplant mothers. The mean concentration of cyclosporine in the colostrum was 22.40 ± 9.43 mcg/L, and the estimated mean daily dose of the drug was 1049.22 ± 397.41 ng/kg/24 h. Only three metabolites (AM1, DHCsA, and THCsA) had mean colostrum amounts comparable to or higher than cyclosporine itself, with the daily doses being 468.51 ± 80.37, 2757.79 ± 1926.11, and 1044.76 ± 948.56 ng/kg/24 h, respectively. Our results indicate a low transfer of cyclosporine and its metabolites into the colostrum in the first two days postpartum and confirm the emerging change to the policy on breastfeeding among post-transplant mothers. A full assessment of the safety of immunosuppressant exposure via breastmilk will require further studies with long-term follow-ups of breastfed children.

## 1. Introduction

Breastfeeding in post-transplant mothers on immunosuppressive therapy was discouraged in the past as the result of the limited data available. However, the American Academy of Pediatrics advises that breastmilk has superior nutrition, especially for preterm infants, who should all receive human milk [1]. The oropharyngeal administration of the mother’s colostrum in preterm infants has recently become a subject of numerous randomized controlled trials to assess the intervention in terms of reducing the incidence of late-onset sepsis and necrotizing enterocolitis, and it is recommended for the routine care of preterm infants in the neonatal intensive care unit [2]. The effect is attributed to a high content of protective immune and trophic biofactors in breastmilk, such as cytokines, lactoferrin, oligosaccharides and antioxidants [3].

Despite promising prospects for the delivery of a healthy baby, pregnancy in a graft recipient remains a high risk with a high incidence of preterm delivery. Maternal and neonatal outcomes in graft recipients are derived mainly from registries, such as the Transplant Pregnancy Registry International (since 1991) or the United Kingdom Transplant Pregnancy Registry (since 1997), or from single-center case series [4,5]. The largest groups are kidney and liver recipients, in whom, since 1963, thousands of pregnancies have been reported worldwide with similar trends in miscarriages, fetal defects, preterm births, low-birth-weight infants, and neonatal deaths. The most recent Transplant Pregnancy Registry International (TPRI) 2018 annual report indicates a high percentage of preterm births below 37 weeks (51% in kidney and 38% in liver recipients), as well as low-birth-weight infants (42% and 30%, respectively) [4]. Underlying pregnancy complications such as preeclampsia and intrauterine growth restriction result in an iatrogenic preterm delivery, often by cesarean section. The TPRI data indicate that its prevalence reaches 50% in kidney, 44% in liver, and 71% in kidney-pancreas recipients [4]. The meta-analyses by Deshpande et al. revealed that 56.9% of kidney and 44.6% of liver recipients deliver by cesarean section [6,7].

As low-birth-weight and preterm infants are prevalent in post-transplant mothers, the well-documented benefits of breastfeeding would be incredibly valuable for them [8]. Therefore, experts consider that breastfeeding in immunosuppressed women might be acceptable on maintenance doses of prednisone (<20 mg/24 h), azathioprine (<2 mg/kg/24 h), cyclosporine A (usual dosage 2–10 mg/kg/24 h), and tacrolimus (usual dosage 0.05–0.2 mg/kg/24 h) [9]. Currently, it is also believed that breastfeeding while on immunosuppressive therapy is probably safe in the case of rheumatic diseases [10,11]. However, in the recent consensus on the management of inflammatory bowel diseases, experts warn that no data are supportive of the use of cyclosporine in breastfeeding because of studies describing the therapeutic blood concentrations of cyclosporine in the breastfed infant [12].

Almost all drugs circulating in maternal blood will be excreted into breastmilk in variable amounts, depending on the molecular structure, lipophilicity, and protein binding capacity [13]. We should also acknowledge the different compositions of breastmilk through the stages of lactation. Lactation has three well-identified stages: colostrum, transition milk, and mature milk. Colostrum is rich in proteins and minerals, immunoglobulins, lactoferrin, and leukocytes and reflects the needs of the newborn during the first week of life. The colostrum expressed by mothers of premature infants is even more abundant in many bioactive components to optimize the intestinal microbiome. The transitional phase lasts from the seventh day up to two weeks postpartum and leads to the establishment of mature human milk. It is a homogeneous mixture with a high content of fats and lactose, with variations in the nutritional components, depending on the stage of lactation, the age of the infant, and individual aspects of each lactating mother [14]. Due to these compositional changes, significant differences may exist in the drug transfer, depending on the time elapsed since the delivery.

On the other hand, the detection of the drug in breastmilk does not mean that it poses a risk to the breastfed baby. When counseling on pharmacologic therapy during breastfeeding, the American Academy of Pediatrics advises balancing the risks for the mother of discontinuing medication with the potential risks for the infant [1]. The experts also note that breastfeeding while continuing therapy administered during pregnancy differs from initiating a new therapy while breastfeeding [15]. As data on breastfeeding while on cyclosporine-based regimens are limited, breastfed infants should be closely monitored for adverse drug effects. Notably, the highest probability of detecting adverse effects due to drug exposure via breastmilk is observed during the first two months of life [16].

While counseling post-transplant patients on breastfeeding, one must take into account multiple factors, weighing the risks and benefits and considering the amount of the drug that is excreted into breastmilk, as well as the extent of oral absorption [17]. According to the latest Transplant Pregnancy Registry International annual report, the rate of post-transplant mothers who breastfeed while on immunosuppression is progressively increasing, and until 2018, there were 519 breastfed infants born to post-transplant mothers identified [4]. Most post-transplant mothers choose not to breastfeed due to fear of prolonged drug exposure for the infant and concerns expressed by their health care professionals [8]. Unfortunately, the available data on breastfeeding safety are limited and are derived mainly from single-center reports. There is still a lack of long-term follow-up studies on the health and development of breastfed children born to post-transplant mothers to monitor growth, immune and kidney function, as well as neurodevelopment. Published reports are not systematical in terms of the measurement of cyclosporine concentration in breastmilk, with different times elapsed from delivery (from a few days to even a few months postpartum), as well as often random times between the drug administration and sample collection. The data supporting the policy for colostrum exposure in the preterm offspring of post-transplant mothers are also sparse.

Moreover, none of the existing evidence takes into account the metabolites of the immunosuppressants that are excreted into breastmilk, which may have partial activity and be responsible for adverse effects.

Therefore, we designed a study to monitor the concentration of cyclosporine and its metabolites in maternal colostrum, aiming to calculate the dose that might be ingested by a breastfed infant.

## 2. Materials and Methods

The study group consisted of seven post-transplant mothers (four kidney and three liver recipients) on immunosuppressive regimens with cyclosporine A and their neonates delivered in the years 2014–2017. All the mothers signed an informed consent form approved by the Institutional Review Board (KB/195/2012).

The recruitment of large groups of patients is always a challenge when post-transplant pregnancies are studied, as they are, in fact, rare events. The most recent Transplant Pregnancy Registry International 2018 Annual Report reports, in total, 3202 pregnancies in graft recipients since 1991 [4]. In our tertiary center, providing care for pregnant solid organ recipients in Poland, we had over 160 deliveries in the years 2000–2019 (including kidney, liver, and individual cases of kidney-pancreas or heart transplants). Therefore, designing a study with large homogeneous groups of post-transplant mothers remains a challenge that would require a multicenter international study design.

This study is a continuation of a previous one that assessed the transfer of tacrolimus and its metabolites in the maternal colostrum.

We collected samples of maternal colostrum, cord blood, and neonatal blood using a similar scheme to that described previously [18]. The colostrum samples were collected from seven participating mothers who were not administered lactation-suppressing drugs. On the first or second postpartum day, they expressed colostrum manually into sterile test tubes (two milliliters) according to the following scheme: t0, directly before the next cyclosporine dose; t1, 2 h after the dose; t2, 4 h after the dose; t3, 6 h after the dose; and t4, 8 h after the dose.

All the samples were deep-frozen and transported to the Institute of Biochemistry and Biophysics of the Polish Academy of Sciences. Materials were secured and stored until the time of analysis in the low-temperature freezer (−80 °C) in the Laboratory of Mass Spectrometry, Drug, and Metabolites Analysis Division.

**Figure 1:**
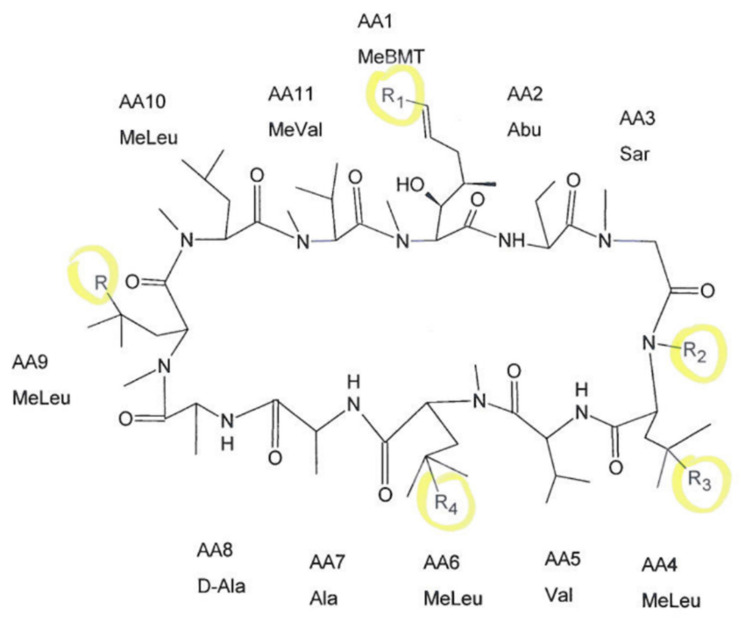
The chemical formula of cyclosporine A (CsA) with marked positions of potential metabolic reactions.

We were able to detect cyclosporine and its monohydroxylated (AM1, AM9) dihydroxylated (DHCsA), trihydroxylated (THCsA), demethylated (AM4N), and demethylated-carboxylated (demCsA) metabolites in the analyzed samples. The chemical formula of CsA and five positions where metabolic reactions may occur are displayed in Figure 1.

Chemicals: Cyclosporin A (CsA), Cyclosporin A-D4 (CsA-D4—used as an internal standard), Cyclosporin AM 1 (CsA AM1), and Cyclosporin AM4N (CsA AM4N) were acquired from Toronto Research Chemicals, Inc. (North York, ON, Canada). All stock solutions were prepared in methanol and stored at −20 °C. Zinc sulfate monohydrate was purchased from Sigma-Aldrich (St. Louis, MO, USA), and analytical grade ammonium acetate was obtained from POCH (Gliwice, Poland). LC–MS grade methanol, HPLC grade methanol, HPLC grade acetonitrile, methyl-tert-butyl ether, and formic acid were obtained from J.T. Baker. Ultra-pure water (Milli-Q water) was produced by a water purification system (Milli-Q, Millipore, Milford, MA, USA).

The sample extraction procedure: A 2 mL colostrum sample (patient samples, calibrators, and quality control samples) was vortexed with 500 µL of 2% aqueous zinc sulfate solution containing an internal standard (CsA-d4) and 500 µL of acetonitrile. To extract CsA and metabolites, 4 mL of methyl-tert-butyl ether was added. Samples were vortexed for 3 min and centrifugated for 10 min at 3000 rpm. The organic portion was transferred into a clean test tube and evaporated under a stream of nitrogen in a water bath Turbo-Vap evaporator (Caliper Life Sciences, Hopkinton, MA, USA). Samples were dissolved in 100 µL of 75% methanol by vortexing for 10 min, and 10 µL was injected into the apparatus. The whole blood samples’ preparation protocol was followed as described previously [19].

Analyses: The instrumentation consisted of a Waters Acquity Ultra Performance Liquid Chromatograph coupled with Waters TQ-S triple-quadrupole mass spectrometer. For the instrument control and data acquisition, Waters MassLynx software was used. Waters TargetLynx was used to process data.

The LC/MS/MS method used has been described previously [20]. The analytes were separated using a Waters BEH C18 column (1.7 µm, 2.1 mm × 50 mm). The mass spectrometer operated in the multiple reaction monitoring (MRM)–positive electrospray ionization (ESI) mode.

The concentration of cyclosporine and its metabolites was calculated using a calibration standard mix derived from a series of calibrator samples by spiking standard stock solution in drug-free whole blood or drug-free colostrum from healthy volunteers. The calibration curves for CsA, AM1, and AM4N were generated by comparing a ratio of the peak area of the analyzed compound to the peak of the internal standard against known analyte concentrations. The patients’ sample was compared with an obtained calibration curve. Mean R2 coefficients of calibration curves from six calibrators were not lower than 0.98. The concentration range in colostrum was 0.5–50 ng/mL, 0.1–50 ng/mL, and 0.1–10 ng/mL for CsA, AM1, and AM4N, respectively. The concentration of AM9 was quantified using an AM1 calibration curve, and the other CsA metabolites’ concentrations were quantified using the CsA calibration curve. To ensure control of the method, in-house control samples were prepared. The method showed good intra-assay and inter-assay precision.

Data on cyclosporine A and its metabolites’ concentrations in colostrum were analyzed to estimate the amounts that would be ingested by a breastfed infant. The dose of cyclosporine and its metabolites was calculated with the use of the AUC curve prepared for measured colostrum levels. The value for t0 was repeated as the value after 12 h (before the next dose). The amounts of cyclosporine and its metabolites excreted into colostrum were calculated as per the protocol described previously [18].

The statistical analysis was performed with the use of the Statistica 13.3 software package (StatSoft Inc., Tulsa, OK, USA). A *p*-value ≤ 0.05 was considered statistically significant.

## 3. Results

The detailed characteristics of the study group are presented in Table 1. The mean gestational age at delivery was 36 weeks, and the mean birth weight was 2839 ± 489 g. All of the deliveries but one were via cesarean section, and the most frequent pregnancy complications included hypertensive disorders and diabetes. The most frequent cyclosporine A daily dose was 200 mg divided into two doses, and the dosages during pregnancy and the early puerperium were titrated according to blood levels to maintain the target level of 120–150 ng/mL (C_0_—before the next dose). None of the mothers chose to breastfeed their infant, and consequently, they were all prescribed lactation-suppressing drugs after sample collection.

The mean concentration of cyclosporine A in the colostrum was 22.40 ± 9.43 mcg/L. With the use of the AUC parameter for colostrum, the daily dose of cyclosporine A per kilogram of body weight was calculated for every infant. It was assumed that each baby would have ingested an amount of colostrum equal to the volume of formula consumed. The estimated mean daily dose of the drug was 1049.22 ± 397.41 ng/kg/24 h.

As for the metabolites of cyclosporine A, we found that only three (AM1, DHCsA, and THCsA) had mean colostrum concentrations comparable to or higher than cyclosporine itself (10.8 ± 5.23, 54.61 ± 29.38, and 19.75 ± 14.2 mcg/L, respectively), with the daily doses being 468.51 ± 80.37, 2757.79 ± 1926.11, and 1044.76 ± 948.56 ng/kg/24 h, respectively.

**Figure 2:**
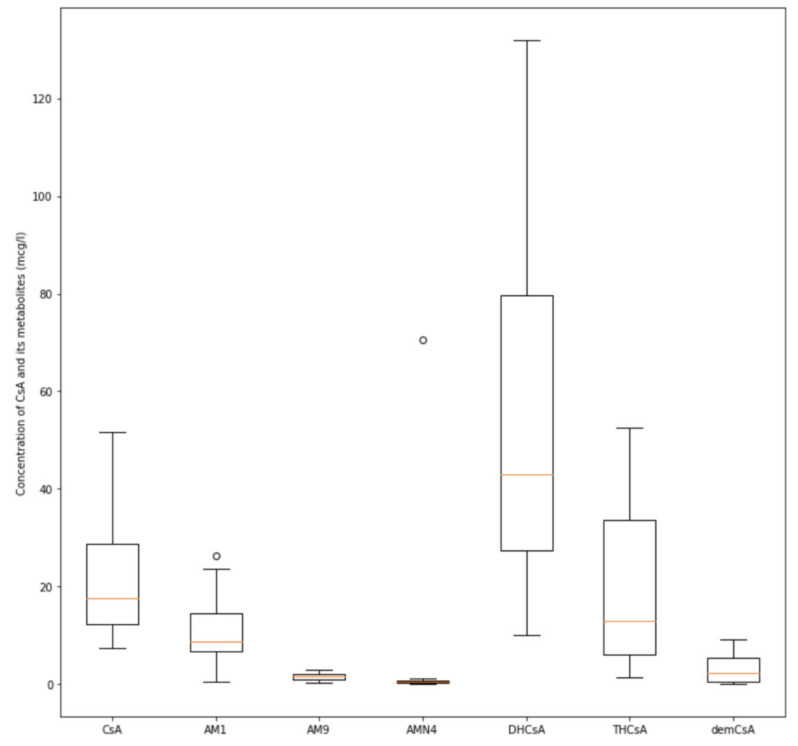
The distribution of cyclosporine A (CsA) and its monohydroxylated (AM1, AM9) dihydroxylated (DHCsA), trihydroxylated (THCsA), demethylated (AMN4), and demethylated-carboxylated (demCsA) metabolite concentrations in colostrum (mcg/L), ° = outlier observations.

**Figure 3:**
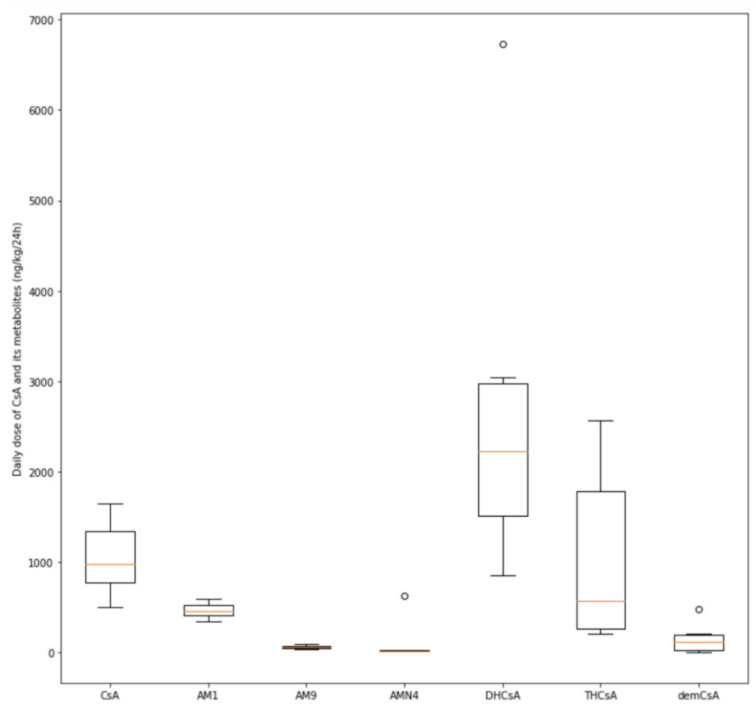
The distribution of daily doses of cyclosporine A (CsA) and its monohydroxylated (AM1, AM9) dihydroxylated (DHCsA), trihydroxylated (THCsA), demethylated (AMN4), and demethylated-carboxylated (demCsA) metabolites per kilogram of body weight (ng/kg/24 h), ° = outlier observations.

The detailed results are presented in Figure 2 and Figure 3.

Due to the heterogeneity and the small group size, it was not possible to analyze the relationship between the subgroups and the parameters of cyclosporine and its metabolites.

## 4. Discussion

The main findings of our study were the calculation of the cyclosporine dose that would be ingested by a breastfed infant in the first days of life and the amount of CsA metabolites transferred to the colostrum. Daily doses were estimated at a level of 1000 ng (0.001 mg) per kilogram of body weight, meaning that the amount of cyclosporine ingested by a breastfed infant would be roughly 1000 times less than the lowest used maintenance dose of 1–2 mg/kg/24 h.

The identified reports on the cyclosporine A–breastmilk transfer are mainly case reports or single-center case series. The concentrations of cyclosporine A were measured mainly in colostrum, as the samples were collected a few days postpartum. The levels reported in the samples obtained from nine women were variable, ranging from 15.5 mcg/L to 465 mcg/L [21,22,23,24,25]. Similarly, when mature milk levels were taken into account, the concentrations (measured in 10 women) were variable, ranging from 46 mcg/L to 596 mcg/L with different periods elapsed from the delivery (from 1 week to 10 weeks postpartum) [21,26,27,28,29]. Our results, with the mean concentration of cyclosporine A in colostrum being 22.77 ± 11.67 mcg/L, fall in the lowest limit of previously published values.

The infant dose that would be ingested with breastmilk was also calculated in a few of those studies, in all of which breastmilk samples were collected over one week after the delivery. In one paper, the calculated infant cyclosporine intake was 6 mcg/kg/24 h [30]. The average intake calculated in a series of three infants was 50 mcg/kg/24 h [21]. However, in two other studies where breastmilk samples were collected from six mothers, the estimated daily infant dose was 100 mcg/kg/24 h [22,26]. The latter estimation means that the breastfed infant would receive around 0.5–1% of the standard cyclosporine maintenance dose (5–10 mg/kg/24 h). On this evidence, it is generally believed that exposition by breastmilk might be acceptable because of the low concentrations of cyclosporine [31]. We estimated that breastfed infants in the first days of life would ingest on average only 1.049 mcg/kg/24 h of cyclosporine, which is an extremely low value when compared to the results mentioned above. The difference might emerge from the fact that the studies mentioned above measured cyclosporine content in mature milk, which is different in composition from that in colostrum.

The transfer of CsA into breastmilk does not necessarily mean that the drug will reach the systemic circulation. One of the most important factors is the oral bioavailability of the medication to the infant. The ingested dose might not be absorbed in the infant’s gastrointestinal tract or be quickly metabolized by the liver (first pass). The extent of CsA oral bioavailability is known only for the adult population, and the amount of drug found in the plasma compartment reaches 28% of the oral dose. The close observation of the infant (gastrointestinal symptoms, signs of liver dysfunction) with monitoring of the infants’ blood levels was suggested for the safe cyclosporine use in breastfeeding mothers [32].

None of the studies assessed the concentration of cyclosporine A metabolites in colostrum. Our results indicate that the dose of metabolites ingested with colostrum would be comparable to cyclosporine A only for three of them (AM1, DHCsA, and THCsA), whereas the others are transferred in trace amounts. Hence, the ingested amount of cyclosporine A metabolites would be at least three orders of magnitude less than the standard pediatric cyclosporine dose.

Cyclosporine metabolites were thoroughly studied in the past [33]. Cytochrome P-350-3A metabolizes the drug, and those reactions result in the formation of over 30 cyclosporine metabolites, mainly by hydroxylation and demethylation. The detection of different metabolites is now possible thanks to the development of modern detection techniques, such as liquid chromatography coupled with tandem mass spectrometry [20]. What seems essential is that selected metabolites may have partial immunosuppressive effects, which may be responsible for the toxic effects of cyclosporine [20]. The metabolites with known immunosuppressive activity are AM1, AM4, and AM4N [33]. Nephrotoxicity was postulated, for example, for AM1 and AM4 metabolites [34]. Notably, impaired kidney function seems to favor the accumulation of cyclosporine metabolites [20]. Those results need further studies, especially taking into account the potential nephrotoxic effects reported for the AM1 metabolite. Our results do not implicate renal impairment in children born to post-transplant mothers, but one should note that none of the mothers chose to breastfeed their infants [35,36].

Among the limitations of our study, we can include the small sample size and the heterogeneity of our study group, consisting of both kidney and liver recipients. The participants agreed to sustain lactation only for the period of sample collection and chose not to breastfeed, so no data from the subsequent weeks of the postpartum period are available. One should note that mature breastmilk is different in terms of composition from colostrum, and therefore, the results might be different when longer breastfeeding is considered. Nevertheless, our results indicate a low transfer of cyclosporine into the breastmilk in the first two days postpartum. Furthermore, we were able to measure the concentrations of cyclosporine metabolites in colostrum. The three metabolites AM1, DHCsA, and THCsA were excreted into breastmilk in comparably low amounts to cyclosporine A itself, whereas the others were detectable in trace amounts only. Therefore, one might assume that their overall effects on the infant are not likely to be of crucial importance.

## 5. Conclusions

As the benefits of mother’s milk are especially pronounced for preterm and low-birth-weight babies, it is evident that the offspring of post-transplant mothers might profit from this intervention. Our results confirm the emerging change to policy on breastfeeding among post-transplant mothers. The short-term follow-up for adverse effects would include monitoring the infant for gastrointestinal symptoms or signs of liver dysfunction, preferably with regular measurement of the infants’ blood levels. A full assessment of the safety of immunosuppressant exposure via breastmilk will require further studies with the long-term follow-up of breastfed children. The safety considerations include but are not limited to immune function disturbances, growth and development impairment, and nephro- and hepatotoxicity, as well as hypertension and hyperlipidemia, which might result in metabolic syndrome development. Preferably, the follow-up should continue into adulthood to see if there are any consequences of immunosuppression exposure, and comparative studies of bottle-fed and breast-fed infants should be carried out to look for differences between in utero only exposure and prolonged exposition due to continued breastfeeding.

## Figures and Tables

**Table 1:** Characteristics of the study group.

	No. = 7
Age of the mothers (years +/− SD)	33.85 ± 3.05
Primipara	3/7 (42.9%)
Mean gestational age (weeks)	36.34 ± 1.59
Premature (<37 weeks)	4/7 (57.1%)
Early preterm (<34 weeks)	1/7 (14.3%)
Birth weight (g +/− SD)	2839 ± 489
Low birth weight (<2500 g)	2/7 (28.5%)
Cesarean delivery	6/7 (85.7%)
Hypertension	5/7 (71.4%)
Diabetes	4/7 (57.1%)
Transplanted organ	Kidney 4/7 (57.1%; Liver 3/7(42.9%)
Years post-transplant (mean +/− SD)	11.33 ± 4.42
Cyclosporine daily dose (mg +/− SD)	195.83 ± 36.56
Other immunosuppressants exposure	Steroids 6/7 (85.7%); Azathioprine 3/7 (42.9%)
